# New Insights into Molecular Mechanisms of Chronic Kidney Disease

**DOI:** 10.3390/biomedicines10112846

**Published:** 2022-11-08

**Authors:** Weronika Frąk, Joanna Kućmierz, Magdalena Szlagor, Ewelina Młynarska, Jacek Rysz, Beata Franczyk

**Affiliations:** 1 Department of Nephrocardiology, Medical University of Lodz, ul. Zeromskiego 113, 90-549 Lodz, Poland; 2 Department of Nephrology, Hypertension and Family Medicine, Medical University of Lodz, ul. Zeromskiego 113, 90-549 Lodz, Poland

**Keywords:** chronic kidney disease, molecular mechanisms, oxidative stress, inflammation, matrix metalloproteinases, nod-like receptor protein 3, gut–kidney axis, novel therapies

## Abstract

Chronic kidney disease (CKD) is a major public health problem with a developing incidence and prevalence. As a consequence of the growing number of patients diagnosed with renal dysfunction leading to the development of CKD, it is particularly important to explain the mechanisms of its underlying causes. In our paper, we discuss the molecular mechanisms of the development and progression of CKD, focusing on oxidative stress, the role of the immune system, neutrophil gelatinase-associated lipocalin, and matrix metalloproteinases. Moreover, growing evidence shows the importance of the role of the gut–kidney axis in the maintenance of normal homeostasis and of the dysregulation of this axis in CKD. Further, we discuss the therapeutic potential and highlight the future research directions for the therapeutic targeting of CKD. However, additional investigation is crucial to improve our knowledge of CKD progression and, more importantly, accelerate basic research to improve our understanding of the mechanism of pathophysiology.

## 1. Introduction

Chronic kidney disease (CKD) is a major public health problem with a developing incidence and prevalence. CKD represents about 10–13% of the population [1]. CKD is an important cause of significant mortality and morbidity in patients worldwide. Unfortunately, both public and clinical awareness remains low [2,3].

CKD is a condition characterized by a dysfunction of the kidneys or its structural abnormalities. It is defined as a glomerular filtration rate (GFR) of less than 60 mL/min/1.73 m^2^, or by the presence of renal marker damage, including albuminuria, urine sediment abnormalities, electrolyte, and other abnormalities due to tubular disorders, structural abnormalities discovered by histology, or imaging and history of kidney transplantation, or both, of at least 3 months’ duration, according to the KDIGO guidelines [4]. CKD can be recognized without acknowledgement of its cause [5].

The risk of CKD increases with age and the condition commonly coincides with hypertension, diabetes mellitus, cardiovascular diseases (CVD), obstructive sleep apnea, or acute kidney injury episodes. In addition, metabolic factors, in particular, insulin resistance, dyslipidemia, and hyperuricemia have been linked with the development and progression of CKD. Some research indicates that CKD is more frequent among men. Notably, African Americans are more prone to kidney damage, compared to Caucasians. Interestingly, there is evidence that low birth weight could predispose people to renal disease. In addition, smoking and excessive use of alcohol and recreational drug intake have been associated with CKD progression (Table 1). However, some risk factors may be modifiable and thus, can delay or even prevent progression to kidney failure [6,7].

Proper treatment of CKD should be considered among all patients in every stage of CKD. Many researchers recommend the evaluation and treatment of patients at the beginning, in order to slow the progression of CKD. Furthermore, it is cost-effective, results in lower patient morbidity, and ensures better outcomes, such as deceleration of the progression of kidney failure and CVD. Moreover, treatment of comorbid conditions is essential [8].

The main consequences of CKD are progression to kidney failure, complications of lessening kidney function, and the development of CVD. Increasing evidence indicates that some of these adverse outcomes can be prevented or delayed by early detection and treatment [6,8]. It is recommended to screen high-risk populations which include individuals with hypertension, diabetes mellitus, and those older than 65 years with blood pressure control, urinalysis, and measurement of serum creatinine with an estimation of GFR [4].

The aim of our work is to describe the new insights into the molecular mechanism of chronic kidney disease. We focused on oxidative stress, the role of the inflammatory cells, neutrophil gelatinase-associated lipocalin, matrix metalloproteinases, the gut–kidney axis, and new treatment targets that have emerged as knowledge about these mechanisms has developed.

## 2. Oxidative Stress

**Figure 1:**
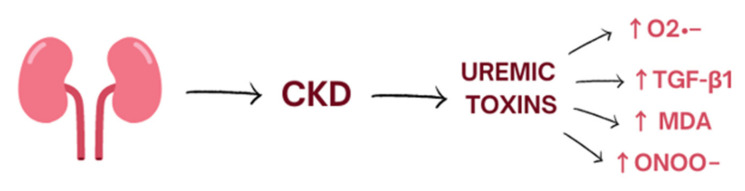
The influence of uremic toxins on the level of oxidative stress. CKD, chronic kidney disease.

Oxidative stress is a process induced mostly by an excessive amount of reactive oxygen species (ROS) and nitrogen species (RNS) and is defined as disturbances in the pro-/antioxidant balance [9]. It leads to a disruption of oxidation–reduction (redox) signaling, molecular damage, and oxidation of biological molecules such as lipids, proteins, and DNA which could lead to covalent crosslinks, single- and double-strand breaks [9]. The kidneys are highly metabolic rate organs, which makes them vulnerable to damage caused by oxidative stress [10]. ROS are mainly produced by the mitochondrial respiratory chain and by enzymes such as NADPH oxidase (NOX1, NOX2, NOX4, NOX5) [11]. Moreover, several uremic toxins are associated with increased oxidative stress in CKD and worsening renal function. Indoxyl sulphate accumulates in the serum of patients with CKD and activates nicotinamide adenine dinucleotide phosphate oxidases (mostly NOX4) which induce cellular production of superoxide. Moreover, indoxyl sulphate enhances production of transforming growth factor-beta1, tissue inhibitor of metalloproteinase-1, proalpha1(I) collagen, free radicals in the cells of vascular smooth muscle, and vascular endothelium [11,12] (Figure 1).

The most important oxidative stress markers in renal dysfunction progress are plasma F2-isoprostanes, 8-oxo-7,8-dihydro-2′-deoxyguanosine, malonyldialdehyde (MAD), advanced oxidation protein products (AOPPs), and carbamylated proteins, as well as asymmetric dimethylarginine (ADMA) and oxidized lipoprotein particles [11].

Elevated levels of oxidative stress have already been found in the early stages of CKD, which is correlated with the progression to end-stage renal disease. The gold standard for the measurement of oxidative stress injury is plasma total F2-isoprostanes, a by-product of lipid peroxidation [13]. Protein carbonyls (PCO) are also valuable biomarkers of oxidative stress which are associated with the state of CKD and their concentration is often higher than that of other biomarkers; however, it is pending whether the carbonylation of proteins have a direct pathological impact or it is a secondary phenomenon [14].

Formation of ROS or changes in ROS production can occur in both the renal cortex and medulla which cause alteration in renal blood flow over sodium/fluid retention to inflammation and fibrotic changes and onset of proteinuria [15].

Oxidative stress has an impact on glomerular injury, albuminuria, interstitial fibrosis, chronic inflammation, and microvascular dysfunction [11]. Moreover, CKD patients with elevated oxidative stress have evidence of autonomic dysfunction correlated mainly with decreased heart rate variability [16]. However, the precise mechanistic pathways through which ROS cause autonomic damage are indefinite. It has been hypothesized that oxidative stress could lead to neuronal injury in a variety of different ways [17].

Albuminuria is one of the most important markers of kidney disease and is an effect of a dysfunctional glomerular filtration barrier with podocyte injury. The mechanism of podocyte injury is complex; however, one of the most significant factors is the vulnerability of podocytes to oxidative damage [16]. The major glomerular sources of ROS are the p47phox-containing NADPH oxidases NOX1, NOX2, and NOX4 [11]. Wang et. al. showed in an experimental model that the deletion of p47phox in mice was correlated with the reduced amount of superoxide and decreased collagen IV production and, as a consequence, protected them from albuminuria and glomerulosclerosis which is characterized by excessive deposition of the extracellular matrix (ECM) [18]. It is worth emphasizing that mitochondrial oxidative DNA damage occurs before podocyte injury and urinary levels of 8-oxo-7,8-dihydroguanosine are independently associated with the incidence of albuminuria [11].

Renal fibrosis, characterized by excessive extracellular matrix deposition, is an important factor in the setting of CKD. The range of tubulointerstitial fibrosis is the best predictor for kidney survival in patients with CKD [19]. The process of renal fibrosis involves a complex interaction between oxidative stress, cytokines, and cell growth factors, especially transforming growth factor-β1 (TGF-β1), which is the key molecule affecting the activation and conversion of fibroblasts to a myofibroblast phenotype [20]. ROS function is similar to TGF-β1: superoxide as a product of NAD(P)H oxidase, also promoting the transformation of fibroblasts to myofibroblasts. Moreover, TGF-β1 increases the activity of NADPH oxidase and the expression of NOX2 and NOX4. P-cresyl sulfate, another uremic toxin correlated with CKD progression, enhances the activity of NOX4-, p22phox-NADPH, and increases ROS production in renal tubular cells. It leads to the reduced cell viability through expression of inflammatory cytokines and profibrotic factors [21]. Together, oxidative stress and TGF-β1, by interacting with each other, promote kidney fibrosis, which leads to chronic kidney damage [22].

Nitric oxide (NO), which is synthesized from arginine by nitric oxide synthase in the endothelium, physiologically inhibits cytochrome C oxidase, a mitochondrial membrane-bound terminal enzyme in the electron transfer chain, and thus reduces the level of oxidative stress. ADMA, which accumulates in the plasma of CKD patients, could block the synthesis of endothelial NO production. In this case, ADMA decreases the amount of NO and consequently increases the production of ROS. As a result, ADMA levels and GFR are negatively correlated [23].

## 3. Inflammation

**Figure 2:**
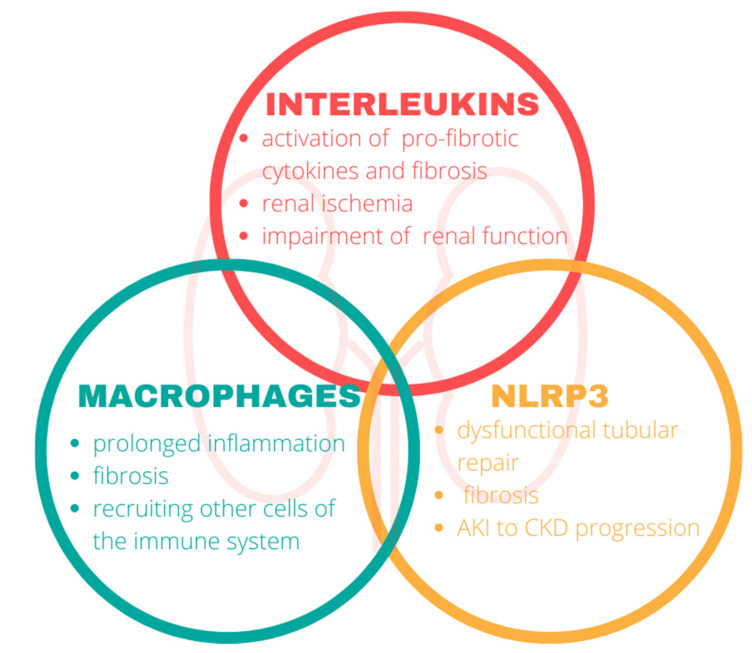
The impact of the immune system on the kidneys. Components of the immune system mediate the disruption of renal functions and play a central role in the progression of chronic kidney disease. NLRP3, nod-like-receptor protein 3.

Inflammation of the kidney involves key components of the immune system. In our review, we focused on the interleukins, macrophages, and nod-like receptor protein 3 in the pathophysiology of CKD (Figure 2).

### 3.1. Interleukins

Interleukins (ILs) belong to a group of cytokines and show various actions in the human body, especially stimulating growth, differentiation, and activation during inflammatory and immune responses [24]. ILs consist of a large group of proteins that can evoke many reactions in cells and tissues [24] Primarily, ILs regulate and mediate inflammatory and immune responses [25]. ILs have autocrine, paracrine, and endocrine influence on other cells. The levels of interleukins in the human body can be used as a diagnostic indicator of the development or progression of many diseases. Below, we discuss the roles of IL-6, IL-1, and IL-20 in CKD pathophysiology [25].

Many studies indicate the pro-inflammatory effects of IL-6 in the pathophysiology of CKD and its role in renal injury. Plasma levels of IL-6 increase with CKD stage, particularly at CKD stage 5 [26]. Furthermore, higher circulating levels of IL-6 are associated with poor outcomes and higher mortality in patients with later stages of CKD [26,27]. Interestingly, it is still unknown if the increased levels of IL-6 constitute a pathogenic trigger or a manifestation of, for example, reduced renal clearance of the cytokine due to the lowered glomerular filtration function associated with renal dysfunction [28]. Additionally, it is worth mentioning that the signaling response of tubular epithelial cells to pro-fibrotic cytokines, such as TGFβ, might be boosted by IL-6. Therefore, IL-6 might be involved in tissue damage and fibrosis [29].

Another interleukin involved in the development of CKD is IL-1. IL-1 is a key player in inflammation, host defense, and acute-phase responses, increasing the infiltration of inflammatory cells and activating the innate immune system [30].

Here, in this study conducted by Bandach et al., in a mouse model of CKD, it is indicated that anemia and renal damage can be modified by the degree of IL-1 expression. The rising level of IL-1 deteriorates kidney function and is associated with a higher degree of renal insufficiency and anemia [31]. That study conducted by Schunk et al. documented that IL-1 regulates tissue accumulation of neutrophils and macrophages, and thereby inflammatory injury in cardiorenal diseases [32]. Ultimately, IL-1 also stimulates fibrosis in kidney tissue [33].

The research data indicated one more interleukin influencing the progression of CKD, which is IL-20. CKD patients at stage 5 expressed significantly higher IL-20 in serum than the control group [34,35]. Chang et al. showed that in the rat models with CKD, IL-20 was highly expressed in the tubular epithelial cells, interstitial immune cells, and glomerular mesangial cells [36]. Moreover, IL-20 stimulated mesangial cells to produce pro-inflammatory mediators and induced apoptosis in tubular epithelial cells. In addition, activation of profibrotic growth factors such as TGF-β1 by IL-20 prompts renal fibrosis. Therefore, IL-20 triggers renal interstitial fibroblasts, and it promotes kidney fibrosis and leads to the progression of renal damage and dysfunction in animal models [36]. It is also worth mentioning that the variability of the IL-20 levels may be associated with the severity of kidney damage in CKD patients; nevertheless, further study is essential [37].

### 3.2. Macrophages

The mononuclear phagocyte system consists of innate immune cells, known as monocytes/macrophages [38]. Monocytes are restrained to the blood compartments, the spleen, and the bone marrow; however, in the presence of inflammation, they are rapidly attracted to the inflamed tissues and undergo macrophage differentiation [38,39]. Moreover, they are essential in host defense against pathogens [39]. Because of their remarkable plasticity and wide range of functional activation stages and phenotypes, macrophages can perform a broad spectrum of specialized roles. Macrophages can secrete a large array of molecules, including producing pro-inflammatory mediators, including IL1, IL6, and tumor necrosis factor (TNF), anti-inflammatory cytokines, such as IL-10, and profibrotic factors, such as TGF-β. There are two types of phenotypes: M1 macrophages that kill pathogens and M2 macrophages that resolve inflammation and tissue repair [38,39].

Macrophage infiltration throughout the kidneys is common in CKD. As a consequence, an increased number of macrophages have been detected in renal tissue in all forms of kidney disease [39]. Furthermore, the macrophages exceed the number of other immune cells such as T lymphocytes, B lymphocytes, Natural Killer, and neutrophils [40]. Macrophages exacerbate the acute kidney injury (AKI) inflammatory response, together with associated cytotoxic effects by generating proinflammatory cytokines [41]. It is also worth noting that they have been identified throughout the whole structure of the kidney, including the glomerulus and the interstitium of the kidney cortex and medulla [42]. M1 macrophages are present in the early phases of inflammation and, as this process goes forward, M2 macrophages trigger repair and fibrosis. As evidenced by the results of studies conducted in research models in rats, M1/M2 macrophage balance could determine CKD development [43].

### 3.3. Nod-Like Receptor Protein 3

NLRP3 is a protein complex and is one of the pivotal components of the immune system [44]. In response to various stimuli of cellular damage, NLRP3 prompts the secretion of proinflammatory cytokines via caspase-1 activation and induces programmed cell death through pyroptosis [45]. The NLRP3 is essential for host immune defenses against infection; however, its dysregulation has been connected to a range of common disorders in humans including CKD [46,47].

Ischemia-reperfusion injury (IRI) is a major cause of AKI and progression to CKD. The literature data suggest the involvement of the NLRP3 in IRI [48]. The study conducted by Zheng et al. showed that there is a link between NLRP3 and failed recovery after AKI. Among mild or severe AKI mouse models, tubular NLRP3 overexpression has been found to be associated with dysfunctional tubular repair, inflammation, and fibrosis. Therefore, an overexpressed amount of NLRP3 was detected in post-AKI kidneys for a long time [49]. According to the literature, prolonged caspase-1 cleavage has been seen in the case of IRI-induced activation of the NLRP3 inflammasome [50]. Comparably, caspase-1, a target of NLRP3, plays a significant part in IRI [51]. Nevertheless, in accordance with this research, endothelial progenitor cells (EPCs) reduced the activation of IRI-induced NLRP3 inflammasome signaling [52]. The role of EPCs is to maintain vascular integrity and stimulate endothelial damage repair. Additionally, they are therapeutic for a majority of kidney disorders; they have been found to decrease the infiltration of immune cells and inhibit fibrosis [52]. Calcium carbonate crystal deposition is a key player in the mechanism of tubular injury and fibrosis. Anders et al. found that calcium crystal deposition activates the NLRP3 [53]. What is more, this study performed by Liu et al. demonstrated NLRP3 activation and cytokine release due to albumin stimulation [54].

As mentioned above, a full understanding of the mechanism of the inflammasome in kidney disease may help to comprehend the pathogenesis of renal disease and NLRP3, which may be a promising therapeutic target in CKD.

## 4. Neutrophil Gelatinase-Associated Lipocalin

Neutrophil gelatinase-associated lipocalin (NGAL) is a glycoprotein that belongs to the lipocalin family [55]. NGAL is expressed at very low levels. However, it is significantly upregulated in injured epithelial cells [56]. Thus, NGAL is a marker of tubular damage [55]. NGAL production from tubular cells may reflect the entity of active renal damage that underlies the chronic impairment condition [57]. NGAL can be produced by kidney tubular cells in response to different types of injuries. It is a recently discovered nephropathy marker. Even in the presence of CKD, NGAL has been proposed as an AKI biomarker. NGAL was elevated particularly in individuals who progressed rapidly to end-stage renal failure [58]. Additionally, it strongly correlates with albuminuria [59]. NGAL is currently recognized by numerous studies as a diagnostic biomarker for AKI [60,61,62,63]. Viau et al. observed that renal NGAL protein content significantly correlated with NGAL excretion, suggesting that the kidney is the major source of urinary NGAL [58]. In response to damaged renal epithelial cells, it quickly induces the production of NGAL. It is worth mentioning that Marouf et al. established that the plasma level of NGAL strongly correlates with albuminuria [64,65].

Accordingly, a crucial role of NGAL in regulating the progression of CKD and cyst formation has been established. Epidermal growth factor receptors (EGFR) are the transmembrane receptors, which belong to the receptor tyrosine kinase family. The role of EGFR is the regulation of ion homeostasis and tubular function. Importantly, acute activation of the EGFR has a positive effect on the repair of the epithelium. On the contrary, chronic activation is deleterious and may induce cyst formation, fibrosis, or even carcinogenic transformation [66]. Furthermore, NGAL mediates the proliferative effect of EGFR in renal tubular cells, following its role in cell proliferation in cystogenesis [58,67].

Additionally, investigations on the role of NGAL in the pathophysiology of kidney injury might result in the identification of novel strategies for treating patients with CKD.

## 5. Matrix Metalloproteinases

Matrix metalloproteinases (MMPs) are proteolytic enzymes involved in extracellular matrix deposition, cell differentiation, angiogenesis, inflammation, proliferation, vascular damage, and apoptosis. There are about 20 mammalian MMPs that are divided into collagenases, gelatinases, stromelysins, matrilysins, membrane type, and other MMPs [68]. MMPs play a significant role in the pathogenesis of many pathologic processes in the multiple afflictions such as rheumatoid arthritis, periodontal diseases, metastases, metabolic diseases of bones, cardiovascular diseases, sterile corneal ulceration, dystrophic epidermolysis bullosa, chronic obstructive pulmonary disease, and emphysema [69]. Different MMPs appear in a series of phases of CKD development such as inflammation, fibroblast/myofibroblast activation, matrix deposition, and fibrosis (Table 2) [68].

Many researchers suggest that the progression of CKD could be related to MMP-7-induced alterations in the extracellular matrix [69]. MMP7 may be more crucial factors than other MMPs in the pathogenesis of kidney diseases [70]. Tan et al. indicated that injection of an expression vector of MMP7 caused passing proteinuria in normal mice. Furthermore, elimination of MMP7 protected mouse against proteinuria and glomerular injury [71]. Liu et al. reported the need for developing novel biomarkers for early detection and prognostic assessment of kidney disorders [72]. They highlighted that the level of MMP-7 in the urine can be used as a potential non-invasive biomarker of kidney disease [73]. Moreover, some researchers have indicated the usefulness of urinary MMP-7 as a predictive factor of acute kidney injury [74]. Zhou et al. found a positive correlation between the level of urinary MMP-7 and renal fibrosis scores and an inverse association with renal function; therefore, urinary MMP-7 levels may serve as a noninvasive biomarker for kidney fibrosis [75].

MMP-9 creates collagen and both of them are chemotactic for neutrophils, stimulating them through cytokines such as interleukin-1 β, and tumor necrosis factor-α to release more MMP-9 [76]. MMP-9 levels are directly correlated with the degree of proteinuria—the main sign of kidney damage that accelerates the progression of CKD toward end-stage kidney disease and also significantly increases cardiovascular risk [77].

Peiskerova et al. revealed that levels of MMP-2 were significantly higher in CKD patients in stages III to V as against those with stages I and II. High levels of MMP-2 are correlated with fibroblast growth factor 23 [78].

MMP-2 is a significant factor which is responsible for glomerulosclerosis development because mesangial cells with high expression of MMP-2 have greater proliferative abilities. Paradoxically, because of increased cell proliferation, MMP-2 can promote ECM production and accumulation in kidney cells through MMP-2, a gelatinase, which physiologically denatures collagen (gelatin) and type IV collagen. Moreover, MMP-2 leads to E-cadherin loss and as a result leads to kidney fibrosis [78].

Both gelatinases, MMP-2 and MMP-9, are produced by glomerular and tubular cells. Researchers have shown that MMP-2 and MMP-9 activation lead to mechanisms that subsequently cause tubular atrophy and fibrosis, and promote inflammation and imbalances in the extracellular matrix as well [79,80].

## 6. Gut–Kidney Axis

As discussed above, chronic kidney disease is characterized by elevated levels of uremic toxins, systemic inflammation, and increased markers of oxidative stress. However, recent studies have shed light on the significant role of the microbiome in CKD. It has been shown that the gut–kidney axis and microbiota act in a reciprocal way. The microbiome is known for its ability to modify many pathways which affect the course of CKD—such as inflammatory, endocrine, or neurologic. On the other hand, CKD has a great impact on the composition of gut microbiota.

The community of intestinal microbes consists of about 80 trillion bacteria and more than 100 bacterial species [81,82]; however, most of them belong to four bacterial phylotypes: Bacteroidetes, Firmicutes, Proteobacteria, and Actinobacteria [83].

Gut microbiota is an ecosystem that affects our physiology in multiple ways. It provides mainly trophic and protective functions [84]. Strengthening the intestinal epithelial barrier by commensal bacteria is one of them.

Rysz et al. [85] reported that dysbiotic microbiome aggravates the advancement of CKD due to the massive accumulation of toxic metabolites in blood. This pathomechanism is mainly caused by the ability of the bacteria to ferment proteins. The result of this process is the formation of urea solutes such as indoxyl sulphate, p-cresyl sulphate, phenyl sulphate, cholate, hippurate, dimethylglycine, γ-guanidinobutyrate, glutarate, 2-hydroxypentanoate, trimethylamine N-oxide, and phenaceturate [86]. Impairment of the epithelial barrier can intensify the translocation of the bacterial-derived uremic toxins and lead to an oxidative stress injury of the kidney. It has been proven that uremic patients often demonstrate endotoxemia despite the absence of clinical infection [87].

Wang et al. [88] have shown excessive colonization of the *Klebsiella* spp., *Proteus* spp., *Escherichia* spp., *Enterobacter* spp., and *Pseudomonas* spp. in patients with ESRD (end-stage renal disease). Moreover, detection of plasma D-lactate level revealed disruption of the gut barrier. As a consequence, bacterial DNAs were detected in the blood of 20% of the ESRD patients. Another study has shown that the stool of these patients was much richer in bacteria such as Enterobacteriaceae (especially Enterobacter, Klebsiella, and Escherichia), Enterococci, and Clostridium perfringens than in healthy persons [89]. In addition, Vaziri et al. [90] reported several histological abnormalities in the hemodialysis population-postmortem examination revealed esophagitis, gastritis, duodenitis, enteritis, and colitis.

The substrate of gut dysbiosis in CKD may be the uremic milieu. Changes in pH in the intestinal lumen lead to disturbance in the composition of the intestinal microbiome. Bacterial urease hydrolyses urea, which is particularly elevated in CKD. The products of this reaction are ammonia and ammonium hydroxide, which contribute to an increase in pH [91]. Modifiable factors that also affect the gut microbiota are low intake of dietary fibre and many medications, such as iron, antibiotics, or phosphate binders [92].

## 7. New Targets of Treatment

Due to the growing knowledge of the molecular mechanisms of CKD, new therapeutic possibilities have emerged. On the other hand, the search for therapeutic targets for renal inflammation is hindered by an incomplete understanding of the pathophysiology.

Based on the inflammatory mechanism of CKD development, we can distinguish two medications that directly target IL-6 ligand and block classical signaling and trans-signaling; they are called sirukumab and siltuximab. In addition, there are antibodies such as tocilizumab and sarilumab whose role is blocking all three types of IL-6 signaling (Table 3) [28]. Another antibody was described by Hsu et al. [93]. They reported that treatment with anti-IL-20 (7E) resulted in a decrease in blood glucose and glomerular area in mice with diabetic nephropathy. Furthermore, improvement in kidney function was observed.

Non-inflammatory mechanisms of CKD are associated with initially dramatic increases in glomerular permeability which consequently leads to proteinuria or proliferation. It is well known that proteinuria is a result of podocyte loss. Damage over 40% of podocytes has many serious consequences such as mesangial expansion, adhesions, focal segmental glomerulosclerosis, or global sclerosis [94]. Therefore, therapeutic strategies targeted toward altering podocyte function will probably be advantageous in these glomerular diseases, which are characterized by nephrotic non-inflammatory glomerulonephritis.

Renal fibrosis is a complex process associated with a network of cytokines/chemokines, growth factors, adhesion molecules, and signaling processes [95]. Studies by Moon et al. [96] demonstrated the therapeutic potential of modulating TGF-β signaling in progressive renal fibrosis. Results of their research indicate that molecular targeting of the transforming growth factor-beta1 signaling pathway can markedly suppress renal injury resulting from unilateral ureteral obstruction. IN-1130, ALK5 inhibitor, is a promising therapeutic agent to suppress or mitigate progressive renal fibrosis.

The above examples are only some of the emerging new targets for the treatment of CKD, and many of them still require additional research.

## 8. Conclusions

The pathogenesis of CKD is complex and many molecular mechanisms play a significant role in the development and progression of the disease. Moreover, it is worth remembering that all the above factors interact, favoring the faster progression of the disease. Inflammation and oxidative stress are inseparable elements that promote kidney fibrosis and loss of function, as shown in the example of interaction between oxidative stress, cytokines, and cell growth factors, especially TGF-β1, which is the key molecule affecting the activation and conversion of fibroblasts to a myofibroblast phenotype. Chronic, low-grade inflammation is a critical component of CKD and plays a crucial role in its pathophysiology.

It is essential to understand molecular interactions between inflammation, oxidative stress, MMPs and other factors for a better understanding of the process of CKD and to find new targets for treatment.

## Figures and Tables

**Table 1:** Risk factors for chronic kidney disease [5,6,7].

Modifiable	Not Modifiable
Smoking	Old age
Nephrotoxins (e.g., alcohol, drugs)	Male gender
Obesity	Family history
Other comorbidities (e.g., hypertension, diabetes mellitus, CVD)	Low birth weight
Metabolic factors (insulin resistance, dyslipidemia, and hyperuricemia)	A non-Caucasian ethnicity

**Table 2:** Pathophysiological mechanisms in CKD of different MMPs are divided into groups [68].

MMP	Group of MMP	Pathophysiological Mechanisms in CKD
**MMP-2, MMP-9**	Gelatinases	Cell proliferation and apoptosis E-cadherin loss Tubular basal membrane degradation Matrix deposition Kidney fibrosis Vascular calcification
**MMP-7**	Matrilysins	Cell proliferation and apoptosis E-cadherin loss Tubular basal membrane degradation Matrix deposition Kidney fibrosis
**MMP-3**	Stromelysins	Cell proliferation and apoptosis E-cadherin loss Tubular basal membrane degradation Matrix deposition Kidney fibrosis Vascular calcification
**MMP-14**	Membrane-type MMPs	E-cadherin loss Tubular basal membrane degradation Kidney fibrosis Vascular calcification

**Table 3:** Mechanism of individual medications.

Medications	Mechanism
Sirukumab and siltuximab	Blockage if the classical signaling and trans-signaling by targeting IL-6
Tocilizumab and sarilumab	Blockage of all 3 types of IL-6 signaling

## Data Availability

The data used in this article are sourced from materials mentioned in the References section.

## Abbreviations

ADMA	Asymmetric dimethylarginine
AKI	Acute kidney injury
AOPPs	Advanced oxidation protein products
CKD	Chronic kidney disease
CVD	Cardiovascular diseases
DFO	Desferrioxamine
ECM	Extracellular matrix
EGFR	Epidermal growth factor receptors
EPCs	Endothelial progenitor cells
ESRD	End-Stage Renal Disease
GFR	Glomerular filtration rate
MAD	Malonyldialdehyde
MMPs	Metalloproteinases
NGAL	Neutrophil gelatinase-associated lipocalin
NO	Nitric oxide
PCO	Protein carbonyls
ROS	Reactive oxygen species
TGF	Transforming Growth Factor
TNF	Tumor Necrosis Factor
